# Periprosthetic knee infection by *Mycobacterium bovis* and *Candida guilliermondii* in the context of a zoonosis: a case report and review of the literature

**DOI:** 10.1186/s13256-019-2009-8

**Published:** 2019-03-08

**Authors:** Emanuel Kuner, Jens Arne Jöckel, Rene Orler, Reto Nüesch

**Affiliations:** 1 Departement Wolhusen, Luzerner Kantonsspital, Spitalstrasse 50, 6110 Wolhusen, Switzerland; 2 Tellklinik Schwyz, Gotthardstrasse 62, 6438 Ibach, Switzerland; 3 Spital Schwyz, Waldeggstrasse 10, 6430 Schwyz, Switzerland

**Keywords:** *Mycobacterium bovis*, *Candida guilliermondii*, Periprosthetic joint infection, Two-stage reimplantation, Zoonosis

## Abstract

**Introduction:**

Periprosthetic joint infections are a major challenge for treating physicians. Musculoskeletal infections with *Mycobacterium bovis* are extremely rare, with an assumed incidence of 0.08–0.1%. Consequently, periprosthetic joint infections with *Mycobacterium bovis* are even less frequent. Fungal periprosthetic joint infections are very rare. No cases of *Candida guilliermondii* infection of implanted prostheses are described in the literature.

**Case presentation:**

An 87-year-old Swiss man with German ethnic origin suffered from symptoms of osteoarthritis of the knee. We present the first described case of periprosthetic joint infection after total knee arthroplasty by both *Mycobacterium bovis* and *Candida guilliermondii* in the context of a zoonosis with 14 months of follow-up. The infection was presumed to originate more than 55 years earlier, when these infectious agents were still present in cattle in Switzerland. After diagnosis of the pathogens, our patient was successfully treated with tuberculostatic and mycocide medication, and a two-stage revision knee arthroplasty was performed. The medication was given for 1 year*.*

The postoperative course was normal and he achieved ambulant musculoskeletal rehabilitation. After 14 months of follow-up no further complication emerged. At all routine consultations, there were no indications for joint inflammation, wound healing was normal, and the range of motion was flexion/extension 110/0/0°.

**Conclusions:**

We found no comparable cases in our literature search. Only a few joint infections by *Mycobacterium bovis* after intravesical instillation of Bacillus Calmette–Guérin are described. Primary infections without previous Bacillus Calmette–Guérin injection appear to be even less frequent. In cases where mycobacterial infection cannot be ruled out, we recommend cultivating mycobacteria cultures for weeks. In addition, a histological examination of the tissue should be carried out. After diagnosis, the concept of a two-stage reimplantation of total knee arthroplasty with mycostatic therapy for 1 year and antimycotic therapy appears to be effective*.*

## Introduction

Periprosthetic joint infections are a major challenge for treating physicians. An incidence of infections of 0.7–1.1% after total knee prosthesis is reported in the literature [1]. Identification of the infectious agent is of major importance for successful therapy. Musculoskeletal infections with *Mycobacterium bovis* are very rare, with an assumed incidence of 0.08–0.1% [2, 3]. We found descriptions of only three cases of periprosthetic joint infection after natural *M. bovis* infection [3–5]. In addition, some prosthetic joint infections were described after intravesical instillation with Bacillus Calmette–Guérin (BCG) as part of the therapy for urothelial carcinoma [6]. To the best of our knowledge, a natural infection with *M. bovis* of a knee prosthesis has not been previously described. Fungal infections of prostheses are also extremely rare [7, 8]. Individual cases with destructive osteoarthritis caused by *Candida guilliermondii* were treated with total knee arthroplasty (TKA) after antimycotic therapy [8]. To date, no cases of periprosthetic joint infection with *C. guilliermondii* have been described in the literature.

## Case presentation

An 87-year-old man from Switzerland with German ethnic origin suffered from symptoms of osteoarthritis of the knee. Preoperatively, there was no suspicion of infectious arthritis. The typical symptoms of osteoarthritis of the knee were present. A routine laboratory test regarding infection parameters (leukocytes, erythrocyte sedimentation rate, C-reactive protein) was without pathological findings. He is a farmer. He grew up on a farm and lived there all his life. He had consumed raw (unpasteurized) milk for years. There was daily contact with animals including cattle. A history of BCG vaccinations was negative. A trip abroad during which an infection could have occurred could be excluded.

**Fig. 1:**
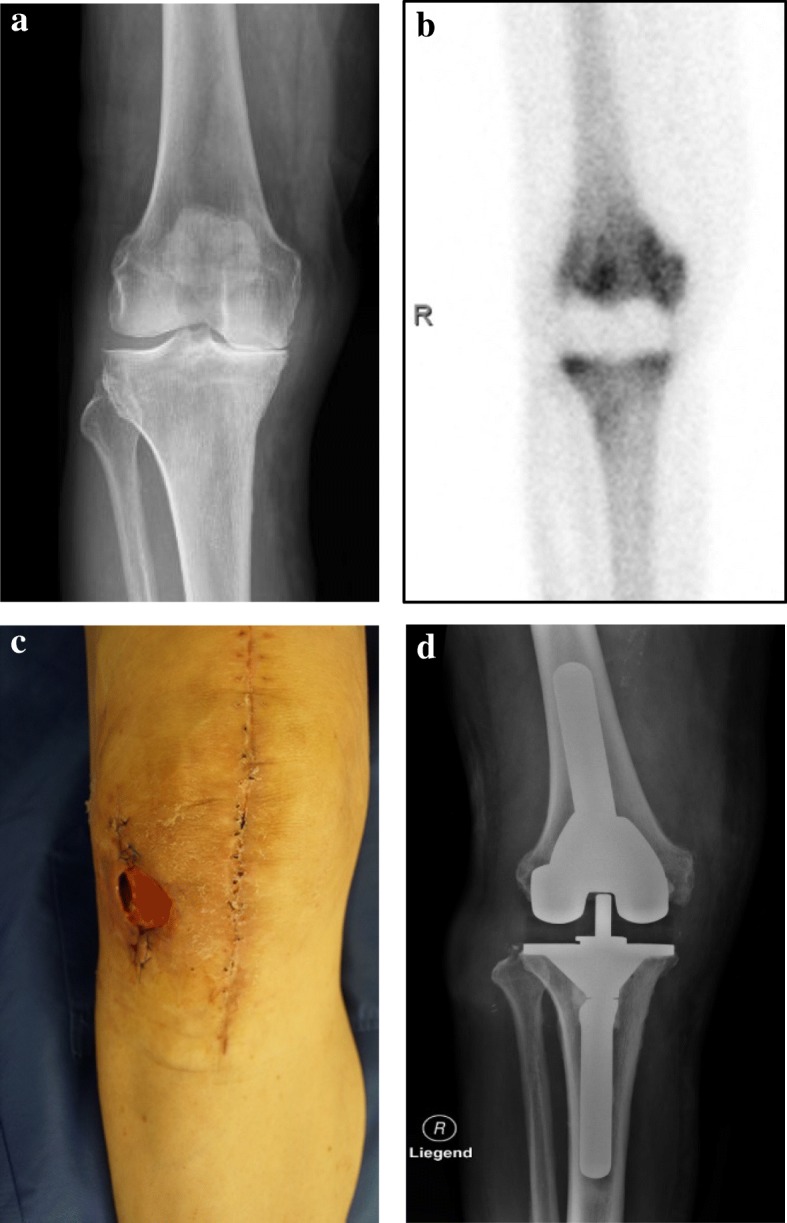
**a** Preoperative X-ray of the right knee showing osteoarthritis of the knee. **b** Following the second revision, a bone scintigraphy (Tc^99^m) was performed. This demonstrated increased levels of radiotracer uptake in the distal femur. **c** The patient developed a wound healing disorder, with a fistula anterolateral to the operational area. **d** Ten weeks after prosthesis removal, reimplantation involving revision total knee arthroplasty was performed under ongoing antimycobacterial medication

The diagnosis was clinically and radiologically confirmed (Fig. 1a). He had chronic obstructive pulmonary disease (COPD) stage II and atrial fibrillation, and was diagnosed as having deep vein thrombosis some years earlier. No malignant disease or immunodeficiency was known. In November 2014, a TKA was performed: implant, Mathys (Bettlach, Switzerland) balanSys®, Femur D (cemented), Tibia 80 (cemented), Polyinlay 8 mm MB rotating. The initial postoperative course was normal. Our patient was discharged from hospital after 8 days. We observed persistent swelling of the knee and persistent wound scab. An aspiration was performed in February 2015, the routine culture was sterile. Two superficial wound debridements were performed in March and April 2015. Following the second debridement, *Staphylococcus epidermidis* and *Corynebacterium* were identified. Antibiotic therapy with co-trimoxazole was initiated, there being no antibiotic-free interval. Due to ongoing wound secretion a third wound debridement was performed in May 2015. A defect of the joint capsule was found. We assumed a prosthetic joint infection starting from the wound healing disorder and undertook a one-stage knee replacement. Intraoperatively, there was no osteolytic bone lesion. Taking into account the expected bacterium and considering existing prosthetic material, antibiotic therapy with vancomycin was started. Tissue samples were obtained and prosthesis sonication was performed. Coagulase-negative staphylococci were detected, and rifampicin (RMP) and clindamycin were given for 3 months. The wound healed after another superficial revision. Eventually, a subcutaneous seroma occurred. Several aspirations were performed, yielding high cell counts (up to 13,000/ml, ≥ 85% neutrophil granulocytes), while routine culture was sterile. Because of the persisting effusion and inflammation, a scintigraphy was performed, which demonstrated enhancement in his distal femur (Fig. 1b).

Because of the persistent inflammation, we decided to perform a knee replacement arthroplasty in two stages. The prosthesis removal was successful and a usual gentamycin Palacos® spacer was implanted in September 2016. We performed tissue sampling and sonication of all implant parts. The materials taken intraoperatively were among others cultured in a liquid medium (BACTEC™ MGIT 960) and on solid media (Löwenstein-Jensen, Middlebrook 7H10-Selective 7H11). The cultures were incubated for 8 to 16 weeks. *M. bovis* was detected in tissue samples. A resistance test was performed for the mycostatic drugs isoniazid (INH), RMP, ethambutol (EMB), and pyrazinamide (PZA). Moreover, *C. guilliermondii* was found by implant sonication. Consequently, the antibiotic therapy was adapted and our patient received RMP, INH, and EMB for 3 months, together with fluconazole for 6 weeks because of the *Candida* infection. After implant removal, he developed a wound healing disorder, with a fistula anterolateral to the operational area (Fig. 1c). Therefore, we performed coverage by a pedicled gastrocnemius flap using a split-skin graft. During the above-indicated defect coverage, tissue samples were again collected. Neither *Mycobacteria* nor *Candida* were detected.

Ten weeks after prosthesis removal, reimplantation was performed (Fig. 1d) under ongoing antimycobacterial drugs, involving revision TKA: Mathys (Bettlach, Switzerland) balanSys® REVISION, Tibia Stem 140 mm 18 mm Offset 4/11, Tibia Plateau size 75, PE Inlay 18, Femur Stem 140 mm 20 mm, Size C, REV Augmentation 10 medial. We stopped antimycotic treatment after negative culture results. The duration of tuberculostatic therapy was maintained 1 year from revision with RMP, INH, and EMB.

The postoperative course was normal and he achieved ambulant musculoskeletal rehabilitation. After 14 months of follow-up no further complication emerged. At routine follow-up consultations, there were no indications for joint inflammation, wound healing was normal, and the range of motion was flexion/extension 110/0/0°. (For timeline compare Table 1.)

**Table 1:** Timeline from his episode of care

11/2014	Total knee arthroplasty due to osteoarthritis
5/2015	Ongoing wound healing disorder in the joint. A one-stage replacement of the total knee arthroplasty was performed. Coagulase-negative staphylococci were detected, rifampicin and clindamycin were given for 3 months
9/2016	Because of the persistent inflammation, the prosthesis was removed. Detection of *Mycobacterium bovis* in the tissue samples. Moreover, *Candida guilliermondii* was found. Antimycobacterial drugs (rifampicin, isoniazid, and ethambutol) and fluconazole were given.
11/2016	Reimplantation of a revision knee prosthesis under ongoing antimycobacterial drugs after 10 weeks.
11/2017	Tuberculostatic therapy was stopped.
01/2018	Fourteen months after revision surgery no further complication had emerged.

## Discussion

Our patient lives in rural surroundings in central Switzerland. Close contact with animals as well as the drinking of raw (unpasteurized) milk must be assumed. Switzerland is supposed to have been free from *M. bovis* since 1960. Our patient was 87-years old. We searched the literature using PubMed and Google Scholar without limitation of the year of publication. In our literature review, we found only a few reported cases of bone and joint infections through a particular *M. bovis* strain termed BCG. Bladder instillation of BCG is a possible treatment for superficial bladder carcinoma. Gomez *et al.* [6] reported one case of joint infection and found another four cases after BCG injections. Our patient did not receive any BCG treatment. Therefore, we postulate a zoonotic disease with an infection before 1960, and reactivation of the latent disease after implantation of the prosthetic joint. Because of the different pathogenesis, we shall not consider the complications occurring after BCG injections here.

To the best of our knowledge, there are only three reported cases of bone and joint infections by *M. bovis* that were unrelated to BCG injections [3–5]. Dubost *et al.* reported one case of total hip arthroplasty loosening [5]. Leach and Halpin described a single case of total hip arthroplasty infection by *M. bovis* in 1993 [4]. Langlois *et al*. reported the first case in a replaced shoulder joint [3]. It appears that our case is the first reported incidence of an infected TKA.

Retrospectively, we assume that this *M. bovis* infection was responsible from the outset for the complicated course. The initially diagnosed infectious agents could have resulted from contamination. The detection of *Candida* is also highly unusual, and fungal prosthetic joint infections are rare [7]. A chronic sinus tract was probably the cause of the prosthetic joint infection with the yeast. According to our literature search, no cases of a prosthesis infection with *C. guilliermondii* have been described. To date, only isolated cases of osteoarthritis by *C. guilliermondii* are known, which have been treated with TKA after antimycotic therapy [8]. Therefore, it is difficult to assess to what extent the *Candida* infection is associated with the pathogenesis in our patient with this specific mixed infection.

The case shows that delayed identification of a pathogen complicates the course of healing. This is because of the difficulty in cultivating *M. bovis*. Because mycobacteria survive for a considerable time in tissue, we consider a 1-year mycostatic therapy to be necessary. In certain cases where such an infection cannot be ruled out, we recommend cultivating mycobacteria cultures for weeks. In addition, a histological examination of the tissue should be carried out. Retrospectively, histology as well as the established concept of a two-stage reimplantation of TKA in the context of the first revision surgery might have shortened the course.

## Conclusions

In summary, a zoonosis with *M. bovis* is extremely rare, making it difficult to find treatment guidelines with high evidence [3–6]. We found no comparable cases in our literature search. Only a few joint infections by *M. bovis* after intravesical instillation of BCG are described. Primary infections without previous BCG injection appear to be even less frequent. In cases where mycobacterial infection cannot be ruled out, we strongly recommend cultivating mycobacteria cultures for weeks. In addition, a histological examination of the tissue should be carried out. After diagnosis, the concept of a two-stage reimplantation of TKA with long mycostatic and antimycotic therapy appears to be effective, as we could show with this case.
